# Examining Multiteam Systems Across Context and Type: A Historiometric Analysis of Failed MTS Performance

**DOI:** 10.3389/fpsyg.2022.813624

**Published:** 2022-03-10

**Authors:** Lauren N. P. Campbell, Elisa M. Torres, Stephen J. Zaccaro, Steven Zhou, Katelyn N. Hedrick, David M. Wallace, Celeste Raver Luning, Joanna E. Zakzewski

**Affiliations:** ^1^Department of Psychology, George Mason University, Fairfax, VA, United States; ^2^Department of Psychology, Clemson University, Clemson, SC, United States; ^3^Division of Leadership Education and Development, United States Naval Academy, Annapolis, MD, United States

**Keywords:** teams and groups, multiteam systems, performance, entrainment, historiometric method

## Abstract

Multiteam systems (MTSs) are complex organizational forms comprising interdependent teams that work towards their own proximal goals within and across teams to also accomplish a shared superordinate goal. MTSs operate within high-stakes, dangerous contexts with high consequences for suboptimal performance. We answer calls for nuanced exploration and cross-context comparison of MTSs “in the wild” by leveraging the MTS action sub-phase behavioral taxonomy to determine where and how MTS failures occur. To our knowledge, this is the first study to also examine how key MTS attributes (boundary status, goal type) influence MTS processes and performance. We conducted historiometric analysis on 40 cases of failed MTS performance across various contexts (e.g., emergency response, commercial transportation, military, and business) to uncover patterns of within- and between-team behaviors of failing MTSs, resulting in four themes. First, component teams of failing MTSs over-engaged in within-team alignment behaviors (vs. between-team behaviors) by enacting acting, monitoring, and recalibrating behaviors more often within than between teams. Second, failing MTSs over-focused on acting behaviors (vs. monitoring or recalibrating) and tended to not fully enact the action sub-phase cycle. Third and fourth, boundary status and goal type exacerbated these behavioral patterns, as external and physical MTSs were less likely to enact sufficient between-team behaviors or fully enact the action sub-phase cycle compared to internal and intellectual MTSs. We propose entrainment as a mechanism for facilitating MTS performance wherein specific, cyclical behavioral patterns enacted by teams align to facilitate goal achievement *via* three multilevel behavioral cycles (i.e., acting-focused, alignment-focused, and adjustment-focused). We argue that the degree to which these cycles are aligned both between teams and with the overarching MTS goal determines whether and how an MTS fails. Our findings add nuance beyond single-context MTS studies by showing that the identified behavioral patterns hold both across contexts and almost all types of MTS action-phase behaviors. We show that these patterns vary by MTS boundary status and goal type. Our findings inform MTS training best practices, which should be structured to integrate all component teams and tailored to both MTS attributes (i.e., boundary status, goal type) and situation type (e.g., contingency planning).

## Introduction

The past decade of organizational research has seen a surge of studies focused on the effective operation of multiteam systems (MTSs; Zaccaro et al., 2020). MTSs are complex networks of interdependent teams that each have their own proximal goals but work within and across teams toward a shared superordinate goal (Mathieu et al., 2001). These systems offer the benefit of flexibility and responsiveness when adaptation needs arise (Mathieu et al., 2001). MTSs are prevalent in many extreme action contexts, such as military and emergency response. Due to the high-stakes nature of these contexts, the price of failed performance is often exorbitant. This is readily apparent in catastrophic situations with unsuccessful MTSs at their center, such as the 2010 explosion of the Deepwater Horizon oil rig, which resulted in the deaths of nearly a dozen people and the large-scale environmental contamination of the Gulf of Mexico (Rico et al., 2016); the 2003 Columbia shuttle crash that killed seven astronauts (Beck and Plowman, 2014; Shuffler and Carter, 2018); and the three recent collisions of U. S. Navy destroyers, which collectively resulted in the deaths of dozens (Torres et al., 2021).

However, MTSs are also ubiquitous in non-extreme and non-action contexts, such as scientific collaborative efforts, cybersecurity, and strategic business alliances (DeChurch and Marks, 2006; Marks and Luvison, 2012; DeChurch and Zaccaro, 2013; Zaccaro et al., 2016). Failures in these MTSs can also be costly. For example, Zano, a startup that aimed to develop and produce advanced drone technology, ultimately squandered $3.5 M in crowd-funded investment and yielded no viable end product (Harris, 2016). In these exemplar cases, the ineffective coordination and collaboration behaviors of the component teams inhibited resilient MTS performance in the face of challenges and resulted in the system not achieving its distal goal. Given the potential for harmful results from the failed performance of these complex systems, it is critical to continue efforts to understand how to avoid such outcomes.

Extant MTS performance research has provided insight into the mechanisms that facilitate optimal performance for MTSs (Zaccaro et al., 2020). In particular, MTS experimental and case studies have found support for the importance of between-team interactions for successful MTS performance across different types of MTSs (e.g., Marks et al., 2005; Beck and Plowman, 2014; Stoate, 2015; Wijnmaalen, 2015; Rico et al., 2016, 2018; Bick et al., 2017; Schecter, 2017; Schipper, 2017; Schipper and Gerrits, 2017; Wijnmaalen et al., 2018, 2019; Pendergraft et al., 2019; Ziegert et al., 2020; Torres et al., 2021). For example, Beck and Plowman’s (2014) analysis of the Columbia shuttle explosion response effort highlighted the impact of between-team interactions on performance enhancing mechanisms. Their finding was echoed in a series of construction context MTS case studies, wherein Wijnmaalen et al. (2018, 2019) emphasized the threat posed to MTS performance by team-level behaviors that inhibit collaboration between groups (i.e., intergroup behavior; Wijnmaalen et al., 2019). Marks and Luvison (2012) found similar importance for between-team interactions, and handoffs in particular, in a drug development strategic alliance MTS. Most recently, Torres et al. (2021) found that a lack of engagement in between-team behaviors drives MTS performance failure. The overarching theme among findings of these studies is clear: interactions between component teams are critical determinants of optimal MTS performance.

Prior studies of failed MTS performance have been somewhat hampered by typical MTSs’ complexity, not only in the multilevel structure and goal hierarchies of MTSs (Rico et al., 2016, 2018), but also the significant dynamism of MTS coordination processes and the contexts within which they typically operate (Luciano et al., 2018b; Pendergraft et al., 2019). That is, given the inherent difficulties of studying MTSs, a lack of evidence persists regarding the mechanisms that drive MTS performance. With few exceptions, the aforementioned MTS studies largely examined static snapshots of performance. The dynamic nature of MTS performance is inherent and apparent in MTS failed performance—failure is indicated by the system not meeting its superordinate goal over the course of the performance episode. Failed MTS performance often results from functional inefficiencies over time. Thus, as with many aspects of organizational performance, effectiveness may be predicted through monitoring various performance indicators. Though this is seen across MTSs from all contexts, emergency response systems are an ideal exemplar. Successful performance in the firefighting context is based not only on whether a fire is extinguished and victims are safely extracted, but this successful outcome may be predicted by indicators throughout the incident, such as dispatch offering continual time checks to incident command. By implication, an MTS’s performance is not only determined through a static, end-of-situation assessment, but also through the continual within- and between-team actions that facilitate goal achievement throughout the performance episode.

This study embraces the complex nature of MTS performance by conducting a temporal analysis of how failures occur over the course of the action phase of an MTS’s performance episode. Similar to stand-alone teams (Marks et al., 2001), MTSs go through different phases as they work to accomplish their superordinate goal, and these phases recur throughout MTSs’ lifespans, constituting multiple performance episodes of cyclical transition and action phases and behaviors (Shuffler and Carter, 2018). While activities in the transition phase (e.g., strategy development, strategic planning) are critical for goal accomplishment, focus on the action phase of MTS performance allows for identification of the execution-oriented behaviors needed for MTS success, including those transition-like behaviors that occur within this phase.

As such, we leveraged Torres et al.’s (2021) model of MTS action subphase performance and related behavioral taxonomy as a basis for understanding where, within a given action phase, failures occur in MTSs. The primary purpose of this study is to provide nuance to the literature’s current understanding of the mechanisms that underlie MTS performance. Additionally, because this inventory includes both between-team behaviors and within-team alignment behaviors (i.e., behaviors enacted within teams that help align component team goals and activities with between-team processes and the distal goal of the system), we were able to conduct a multilevel examination of behavioral factors that influence MTS performance (see Table 1 for the full taxonomy). Our first research question is as follows:

**Table 1:** MTS Action Subphase Behavioral Inventory.

Within-Team MTS Action Subphases and Behaviors	
Acting	Within-team behaviors associated with goal striving and MTS goal accomplishment, including implementing adaptation plans identified in recalibrating phase
1^*^	Execute protocols that coordinate team members’ activities around MTS goal accomplishment
2^*^	Enact appropriate alignment of team members’ activities to facilitate appropriate pacing of MTS actions
3^**^	Implement modifications to member resources to align with MTS task requirements
4^**^	Implement modifications to team member response sequence to maintain appropriate pacing of MTS task requirements
5^**^	Implement modifications to team member tasks and actions to align with the actions of other focal teams in the MTS
Monitoring	Continuous within-team observation and communication used to track goal progress and quality of MTS performance over time
6^+^	Track team members tasks and progress toward MTS goal accomplishment
7^+^	Track use and availability of team resources that support MTS goal accomplishment
8^+^	Track information about an event/incident/threat impacting MTS goal accomplishment
9^++^	Exchange information with focal team members regarding progress toward accomplishing team and MTS goals
10^++^	Exchange information with team members regarding changes in MTS environmental characteristics and constraints that signal the need for adjustment or adaptation
11^+^	Acknowledge cues and triggers in the MTS environment (e.g., elements of a developing event/incident/threat) that indicates component team member adjustments or adaptation is required for MTS goal accomplishment
12^++^	Alert team members about incidents requiring MTS adjustment or adaptation and the impact on between-team interactions
Recalibrating	Identification of the need for within-team action adjustments and adaptation; development of such plans for subsequent actions; and dissemination of these plans to team members
13	Exchange information with team members to develop a shared understanding of incident triggers and adjustments or adaptation cue stream to appropriately align team members actions with modified MTS actions
14	Develop a course of action for adjusting or adapting team member actions to align with MTS actions
15	Communicate updated team member action and/or between-team action plan(s) with focal team members
**Between-Team MTS Action Subphases and Behaviors**	
Acting	Between-team behaviors associated with goal striving and MTS goal accomplishment, including implementing adaptation plans identified in recalibrating phase
16^*^	Execute interdependence between-team actions according to MTS sequence and timing
17^*^	Engage in between-team back-up behavior, assisting component team task accomplishment
18^*^	Share material and personnel resources between teams
19^**^	Implement modifications to between-team actions and tasks
20^**^	Implement modifications to between-team response sequence to maintain appropriate pacing of MTS tasks
Monitoring	Continuous between-team observation and communication used to track goal progress and quality of MTS performance over time
21^+^	Track component teams’ activities and process toward MTS goal accomplishment
22^++^	Exchange information between teams regarding MTS environmental characteristics and constraints that signal the need for between-team action adjustment or adaptation
23^++^	Exchange information between teams regarding team goal progress
24^++^	Exchange information between teams regarding team resources and constraints
25^+^	Acknowledge cues and triggers in the MTS environment (e.g., elements of a developing event/incident/threat) that indicates between-team action adjustment or adaptation is required for MTS goal accomplishment
26^++^	Alert other teams about incidents requiring between-team adjustment or adaptation
Recalibrating	Identification of the need for between-team action adjustments and/or adaptation; development of such plans for subsequent actions; and dissemination of these plans between component teams
27	Exchange information between teams to develop a shared understanding of incident triggers and adjustment or adaptation cue stream
28	Develop a course of action for adjusting or adapting between-team coordinated action
29	Communicate updated action plan between component teams

Adapted from Torres et al. (2021).

* Aligning-type acting subphase behaviors;

** Adjusting-type acting subphase behaviors.

+ Tracking-type monitoring subphase behaviors;

++ Communicating-type monitoring subphase behaviors.

*RQ1: Where do failures occur within the MTS action phase of the performance episode, and what failure behaviors have the most impact from both within- and between-team levels?*

Early MTS theory was largely built upon controlled laboratory experiments, quasi-experiments, and single-context qualitative studies (e.g., Marks et al., 2005; Lanaj et al., 2013; Allison and Shuffler, 2014; de Vries et al., 2016). Given this, MTS researchers have argued that more studies should explore and compare MTSs across various contexts “in the wild” (e.g., Shuffler and Carter, 2018; Zaccaro et al., 2020). Methodologies closer to the source (e.g., field studies, multiple case studies) are needed to advance nuanced understanding of these complex systems, their performance, and the challenging situations they face. As individuals likely behave differently under the higher pressure and stakes associated with real-world situations, compared to those within a manufactured lab setting, such studies may enlighten intricacies of the processes and mechanisms that drive performance, particularly in stressful scenarios. Extant theory has propelled MTS research forward, lending opportunity for *in situ* studies to apply, test, and validate the knowledge base that has been built to date.

MTSs “in the wild” not only differ from lab-based MTSs based on their contexts, but also as a function of the features of the system (i.e., system attributes), which are often challenging or impossible to vary in laboratory or artificial environments. MTS theory suggests that system attributes are influential for system effectiveness (Zaccaro et al., 2012). For example, Zaccaro et al.’ (2012) typology of MTS attributes noted boundary status as a critical MTS attribute and defined it as whether the component teams embedded in the system come from a single organization (internal) or multiple organizations (cross-boundary or external). This distinction is theorized to be highly influential for MTS inter-team processes (Luciano et al., 2018b; Zaccaro et al., 2020). However, no prior study has empirically examined the impact of this central feature of MTSs on performance.

Another attribute that likely influences system functioning, including the coordinating mechanisms that optimize MTS performance, is goal type (Zaccaro et al., 2020). MTS goal type delineates the nature of the core task associated with the system’s superordinate goal as either physical, involving “physical skills, a linear workflow, applying existing knowledge, and a tangible product,” or intellectual, involving “mental skills, a nonlinear work process, the derivation of new knowledge, and information as the primary work outcome” (Devine, 2002, p. 296). As with boundary status, different MTS goal types should have implications for MTS processes and performance; yet no known prior studies have investigated variance in MTS goal type as a driver of performance. To that end, we aim to respond to these unanswered questions in empirical literature by using historiometric analysis to examine a large set of failed MTS performance cases that vary on these MTS attributes and uncover emergent themes to inform related theory.

Early MTS studies, aware of the high-stakes nature of their typical performance environments, primarily examined mission critical contexts, particularly military MTSs (e.g., Marks et al., 2005; Davison et al., 2012; Lanaj et al., 2013). Recent case studies have included MTSs from varied industries (e.g., strategic organizational alliances, Marks and Luvison, 2012; railways, Schipper, 2017; Schipper and Gerrits, 2017). However, both early and recent case studies have examined MTS performance *within* single contexts rather than *across* multiple contexts. While single-context studies offer deep insight into the specific workings of a particular system, context, or situation, findings are often limited in application to only MTSs with similar attributes and/or comparable situational parameters. Comparatively, cross-context analysis lends breadth and generalizability to findings that more readily inform training applications and offer testable implications for broader theory. Further, when methods include varied MTSs from different contexts, subsequent analyses can offer greater nuance to enlighten underlying performance mechanisms. That is, cross-context study design affords researchers the opportunity to determine whether patterns found are generic and broadly applicable or if they only apply to or change as a function of differences among MTSs. As such, we aim to expand the field’s understanding of MTS performance by leveraging cross-context analysis to examine a large set of cases of MTSs with differing attributes as they fail to perform optimally across differing situations.

Given the multicontext nature of the selected cases, we also answer calls for implementing cross-context study designs (e.g., Mathieu et al., 2018; Zaccaro et al., 2020), allowing for comparative examination of two MTS attributes: boundary status and goal type. That is, a secondary purpose of this study is to illuminate the boundary conditions surrounding the underlying mechanisms of MTS performance. Accordingly, we use historiometric analysis of 40 cases of failed MTS performance across various contexts to explore the second research question:

*RQ2: How do boundary status and goal type influence when and how performance failures occur?*

### MTS Action Subphases

We used Torres et al.’ (2021) MTS action subphase model and behavioral taxonomy, shown in Table 1, to examine the failed MTSs. This framework postulates that three inter-related and cyclical subphases are embedded within the action phase of MTS performance—*acting*, *monitoring*, and *recalibrating*. Each subphase occurs at both the within- and between-team levels. That is, acting, monitoring, and recalibrating behaviors occur within teams to facilitate between-team goal accomplishment (i.e., within-team alignment behaviors), as well as between component teams in the MTS. The MTS action subphase model expands Marks et al.’s (2001) framework of episodic team processes. Further, the model is applied to MTS performance and its inherently adaptive nature, which is important for MTSs as they are ubiquitous in turbulent and non-routine environments (e.g., Mathieu et al., 2001; DeChurch and Mathieu, 2009; Standifer, 2012; Uitdewilligen and Waller, 2012; Zaccaro et al., 2012; DeChurch and Zaccaro, 2013; Kugler et al., 2016). The model dynamics as presented include temporal and reciprocal interactions among the three action subphases. Within these subphases, multilevel processes comprising separate, often simultaneous behaviors occur within and among the component teams of a given MTS. Each subphase becomes increasingly important as the system engages in adaptive processes; therefore, the behaviors that comprise each subphase are critical for optimal MTS performance.

MTS action episodes typically begin with goal-directed behavior (i.e., acting), wherein teams engage in interdependent, goal-directed MTS taskwork (Marks et al., 2005). These activities occur at two levels: (1) within teams to facilitate between-team processes and (2) between teams to work toward higher-order distal goals. Thus, Torres et al. (2021) define MTS acting as the within-team and between-team behaviors associated with goal striving and MTS goal accomplishment, including typical and/or protocol-driven alignment behaviors and adjustment-driven behaviors (i.e., implementing adaptation plans identified in the recalibrating phase; p. 192). Given the bimodal nature of acting behaviors as defined, we considered alignment and adjustment acting behaviors separately to allow for more granular assessment of the behavioral patterns in each of the cases, in line with our goal of adding nuance to existing theory of MTS performance. By distinguishing these types of behaviors within MTS failure cases, we may illuminate subtle differences in the performance mechanisms and the boundary conditions within which they operate. As such, we categorize alignment behaviors as *those involving executing or enacting tasks or activities, often that are prescribed by protocols for team and system goal accomplishment*. Adjustment behaviors are *those that implement modifications to standard tasks or activities, often following recalibration*. See Table 1 for categorization of acting behaviors.

The second subphase, monitoring, is defined by Torres et al. (2021) as continuous within- and between-team observations used to track MTS goal process and progress over time, as well as the communication of these tracked observations. As with acting behaviors, we considered tracking and communicating monitoring behaviors separately. Tracking behaviors comprise *observing and acknowledging the behaviors of other team members and MTS members*, while communication behaviors comprise *the exchange of information about those tracked behaviors or situational changes*. See Table 1 for a categorization of monitoring behaviors.

**Figure 1:**
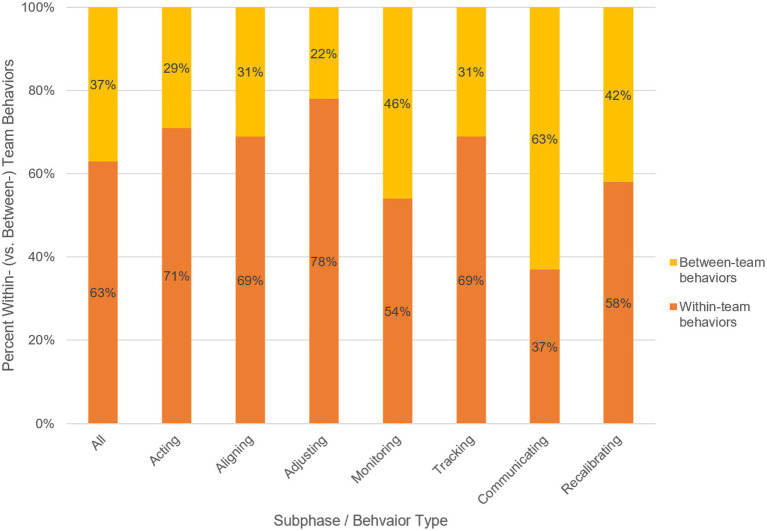
Percent Within vs. Between Behaviors across All Cases by Subphase and Acting and Monitoring Behavior Types. The y-axis represents the percentage of within-team (orange) vs. between-team (yellow) behaviors represented in each behavior type as indicated in the x-axis. The x-axis includes a column for all of the coded behaviors, as well as each subphase behavior type (i.e., acting, monitoring and recalibrating) and each subtype for acting (i.e., aligning and adjusting) and monitoring (i.e., tracking and communicating). For example, the furthest right column indicates that, of all of the coded recalibrating behaviors, 58% occurred at the within-team level and 42% occurred at the between-team level.

Following successful tracking and communication of internal and external triggers (i.e., monitoring), the recalibrating subphase is initiated, and adaptive performance processes are engaged, including the development and dissemination of plans for adjustment and/or adaptation behaviors within and between component teams (Torres et al., 2021; see Table 1 for a delineation of recalibrating behaviors). The full cyclical and iterative process of MTS action phase performance is presented in the MTS action subphase model (refer to Figure 1 on p. 193 in Torres et al., 2021).

Together, MTS performance literature and Torres et al.’s (2021) framework of MTS performance set the stage to tell a complex story of MTS failure. The present study aims to clarify the mechanisms driving MTS performance failures and uncover the impact of MTS attributes (i.e., boundary conditions surrounding the mechanisms). This nuance will aid the development of targeted interventions to optimize MTS performance and the prevention and mitigation of further MTS-related catastrophes. We build upon MTS research theory and themes, leveraging the diagnostic capacity of the MTS action subphase behavioral inventory, to conduct a cross-context examination of 40 cases of MTS performance failures.

## Materials and Methods

Our methodological approach builds upon case study analysis methods used in recent MTS studies (e.g., Pendergraft et al., 2019; Torres et al., 2021), leveraging historiometric analysis (HMA; Simonton, 2003; Crayne and Hunter, 2018) to conduct a structured examination sof MTS performance situations and contexts. In a recent review, Burke et al. (2021) describe historiometry as “special case of archival analysis,” unique for its use of quantification to support qualitative data analysis of historically significant events. This approach works bottom-up to integrate systematic qualitative analysis with supporting quantitative descriptive statistics. HMA is conducted through in-depth, iterative review and coding of archival, narrative data—in this study, these data consist of case studies of MTS performance. The aim of HMA is to uncover emergent themes in the form of descriptive or prescriptive conclusions or propositions, which serve as groundwork for future hypotheses for empirical study.

HMA has been used to examine MTS-related phenomena, including leadership processes (e.g., DeChurch et al., 2011), and is noted to be beneficial for studying teams “in the wild” (Burke et al., 2021). Crayne and Hunter (2018) argue that HMA is most useful when context and situational specifics, unique or rare data samples, and/or longitudinal data are examined, qualities which align with our data. Each MTS failure case represents a specific situation within a certain context that determines which processes and behaviors are observable, emergent, and appropriate. Additionally, many cases represent relatively rare situations, such as space shuttle crashes, active shooter emergencies, and large-scale environmental crises.

In addition to this study design fitting with criteria delineated by HMA experts, Mathieu (2012) has noted the utility of HMA for “comparative study of complex MTS forms” and the ability to “infer cause–effect relationships more so than would have been afforded by a cross-sectional design” (p. 533). Our cases constituted longitudinal data, covering the action phase of performance for each MTS. As such, we answer calls for specificity in targeted analysis of MTS scenarios focused on temporal dynamics (Mathieu, 2012). That is, while the use of archival data has its limitations, it offers benefits when data include temporal aspects that can be leveraged to consider the evolution of behaviors and events within each MTS.

### Case Identification and Inclusion Criteria

Following Crayne and Hunter’s (2018) 10-step HMA process, we began with study design and investigative piloting using a small set of known cases of MTS performance failures (e.g., the explosion of the Deepwater Horizon oil rig, resulting in the deaths of nearly a dozen people and the large-scale environmental contamination of the Gulf of Mexico, reviewed in Rico et al., 2016). Notably, our case identification process began with the intention of including cases of successful and failed MTS performance for comparison; however, data sources appropriate for HMA are more available for failures than successes. We suspect that MTSs often do not publicly document cases of optimal performance for proprietary purposes and that this is particularly true for MTSs in certain contexts and/or with certain attributes (e.g., business MTSs with intellectual goal types). We suggest that cases of failed MTS performance present unique benefits for practitioners and researchers alike, above and beyond the analysis of successful cases, including informing training efforts focused on error management, resilience, and recovery.

The investigative piloting process helped determine the appropriate inclusion criteria for effective cross-case comparison. First, the case had to encompass the work of two or more teams working interdependently toward a common goal, while also having lower order team goals (i.e., constituting an MTS). Second, the event had to have publicly available information on the structure of the component teams within the MTS and its key attributes. Third, the superordinate goal of the MTS had to be readily apparent and not have been achieved (i.e., constituting it a case of failed MTS performance). Finally, the available event information had to be from reputable sources and include detailed information on the team-level behaviors that occurred throughout the action phase of the MTS performance episode. Keeping with the purposes of the study, no criteria related to MTS context or attributes were applied. To ensure saturation of the dataset with various case types and reliability of the analyses, both unique cases (e.g., environmental crises) and multiple cases from contexts where similar failures are somewhat prevalent (e.g., airplane crashes) were included.

Four graduate researchers with knowledge of MTS literature used the criteria and a snowball-like method to uncover archival material on cases of failed MTS performance through Google, Google Scholar, large newspaper websites, and official investigative report databases (e.g., National Transportation and Safety Board, National Institute for Occupational Safety and Health). The researchers identified a particular event or type of event and searched known databases for documentation related to those events. The data source types included scholarly articles, government articles and presentations, legal documentation, organizational documents (e.g., process manuals from the companies represented in the MTSs), news articles, and investigative reports. Notably, the primary data source used for nearly all cases was an investigative report, which was generated by official government agencies and/or independent industry organizations and consisted of descriptive event summaries; details about the organization(s), teams, and persons involved in the case; a timeline of events, including within- and between-team behaviors; and/or quotations from person(s) involved in the case. Though the cases were not all represented by the exact same number of sources or combination of data types, the relative consistency across all cases in the set provides confidence that no confound exists related to the data source type. For a full list of the number and types of data sources used for each case, see Table 2.

**Table 2:** Included Cases of Failed MTS Performance.

Industry	Case summary	Data Source(s)	Bound. status	Goal type
Business/Religion	2018 Willow Creek Church dissolution following sexual harassment scandal response effort	2 industry investigative reports, 3 news articles	E	I
Business/Product Dev.	2015 Zano startup development abandonment	1 investigative journaling report, 1 org document, 1 news article	E	I
Business/Construction	1990s Denver airport automated baggage system implementation abandonment	1 industry investigative report, 1 news article	E	I
Business	2010 Deepwater Horizon explosion and oil spill	6 gov’t/industry investigative reports, 2 gov’t articles, 3 academic articles, 2 news articles, 1 org manual	E	P
Business/Government	1987–1992 DMV records digitization abandonment	1 academic article, 1 legal document, 1 news article	E	I
Business/Government	2013 Affordable Care Act website launch delay	2 gov’t investigative reports, 1 academic commentary article, 7 news articles	E	I
Science/Government	2003 NASA Columbia shuttle landing crash	3 gov’t investigative reports	I	P
Emergency Response	2013 Washington, DC Navy Yard active shooter response effort	2 gov’t investigative reports, 4 news articles	E	P
Emergency Response	2016 Orlando, FL Pulse nightclub active shooter response effort	2 gov’t/industry investigative reports, 1 news article	E	P
Emergency Response	2007 Blacksburg, VA Virginia Tech active shooter response effort	1 gov’t investigative report, 3 news articles	E	P
Emergency Response	2016 Parkland, FL high school active shooter response effort	1 news article, 1 gov’t meeting presentation and synopsis	E	P
Emergency Response	2016 MD firefighter fatality during civilian welfare check	1 gov’t investigative report	E	P
Emergency Response	2013 TX fertilizer plant explosion and response effort	4 gov’t investigative reports, 1 gov’t article, 7 news articles, 5 academic articles	E	P
Emergency Response	2018 PA firefighter fatality during building collapse	1 gov’t investigative report	E	P
Emergency Response	1998 NY firefighter fatality during floor collapse	1 gov’t investigative report	I	P
Emergency Response	2016 DE firefighter fatality during arson response effort	1 gov’t investigative report	I	P
Emergency Response	2017 TX firefighter fatality during arson response effort	1 gov’t investigative report	I	P
Emergency Response	2018 TX firefighter fatality during grass fire response effort	1 gov’t investigative report	I	P
Military	2001 USS Greeneville—Ehime Maru collision	2 gov’t investigative reports, 1 legal document, 3 news articles	I	P
Military	2012 USS Essex—USNS Yukon collision	1 gov’t investigative report, 5 news articles	I	P
Military	1969 USS Evans—HMAS Melbourne collision	1 gov’t investigative report	E	P
Military	2017 USS Antietam grounding	1 gov’t investigative report	I	P
Military	2013 USS Guardian grounding	1 gov’t investigative report, 1 gov’t article, 4 news articles	I	P
Military	2012 USS San Jacinto—USS Montpelier collision	1 gov’t investigative report, 1 gov’t article, 4 news articles	I	P
Military	1975 USS Belknap—USS Kennedy collision	1 gov’t investigative report, 1 gov’t article, 3 news articles	I	P
Transport	2015 Clipper Quito—Lurongyu collision	1 gov’t investigative report	E	P
Transport	2017 Eric Haney grounding	1 gov’t investigative report	E	P
Transport	2013 Amarillo, TX three-train collision	1 gov’t investigative report, 6 legal documents, 1 org manual	E	P
Transport	2016 Granger, WY two-train collision	1 gov’t investigative report, 6 legal documents, 1 org manual	E	P
Transport	2009 Atlantic Ocean, Air France flight 447, in-air crash	1 gov’t investigative report	E	P
Transport	2016 Chicago, IL, American Airlines flight 383, take off abandonment	1 gov’t investigative report, 1 org manual, 1 gov’t article, 1 gov’t meeting presentation and synopsis	E	P
Transport	1996 Quincy, IL two-airplane runway collision	1 gov’t investigative report, 3 gov’t articles, 2 org manuals	E	P
Transport	2005 Teterboro, NJ, Platinum Jet Mgmt., departure crash	1 gov’t investigative report, 4 gov’t articles	E	P
Transport	2015 Taipei, Taiwan, TransAsia flight 235, departure crash	1 gov’t investigative report	E	P
Transport	2016 Dubai, UAE, Emirates flight 521 airplane landing crash	2 gov’t/industry investigative reports	E	P
Transport	2014 Java Sea, AirAsia flight 8,501, in-air crash	1 gov’t investigative report	E	P
Transport	2014 Magong, Penghu Island, TransAsia flight 222 landing crash	1 gov’t investigative report	E	P
Transport	2016 Lagos, Nigeria DANACO flight 992 in-air crash	2 gov’t/industry investigative reports	E	P
Transport	2009 Lubbock, TX Empire Airlines flight 8,284 landing crash	2 gov’t/industry investigative reports	E	P
Transport	2015 Islamabad, Pakistan, Bhoja flight 213 landing crash	1 gov’t investigative report	E	P

Boundary status: E = external, I = internal. Goal type: I = intellectual, P = physical.

From these sources, the researchers extracted information regarding (1) the attributes of the MTSs involved in each event and (2) coherent behavioral chronologies of the MTSs and their component teams. That is, as viable cases were uncovered, the researchers documented the structure and attributes of the component teams and system, including size, team functions, superordinate goal and goal type, and leadership structure, as well compositional (e.g., boundary status), linkage (e.g., interdependence), and developmental (e.g., tenure) MTS attributes (Zaccaro et al., 2012). Finally, the researchers assembled a timeline of events, including the component teams’ and their members’ key behaviors, from the source materials. These two buckets of information comprised the data for each case.

### Coder Training and Coding

In addition to using Torres et al.’s (2021) theoretical model of MTS performance, we leveraged the related behavioral taxonomy of MTS action subphase performance as a basis for understanding where, within a given action phase, failures occur in MTSs. Given the granular nature of the taxonomy (see Table 1 for the full list of behaviors), we aimed to provide nuance to the literature’s current understanding of MTS performance mechanisms *via* focus on failure cases. Because this inventory includes both between-team behaviors and within-team alignment behaviors (i.e., behaviors enacted within teams that help align component team goals and activities with between-team processes and the distal goal of the system), we were able to conduct a multilevel examination of behavioral factors that influence MTS performance. As such, this taxonomy served as a codebook for identifying behaviors in each MTS failure case.

The same four MTS-aware graduate researchers then trained on Torres et al.’s (2021) MTS action subphase behavioral inventory, which provides three types of information: first, whether a within-team alignment behavior or between-team behavior was occurring; second, whether the behavior was within the action, monitoring, or recalibrating subphase; and third, which specific behavior within those subphases was enacted. The researchers coded a subset of 10 cases, at least one from each context represented in the dataset, until acceptable agreement levels (i.e., over 75% agreement) were achieved and maintained. The remaining 30 cases were then coded by rotating pairs of coders to ensure an intact shared mental model of the coding scheme and to mitigate coder fatigue, resulting in an average agreement of 87%. The minimal disagreements that arose after training resulted from context-specific details and were resolved through review and discussion of source materials until consensus was reached.

### Analysis

Following coding, descriptive statistics were calculated for each case, summarizing the counts and proportions of codes at the behavioral, subphase, and between- vs. within-team levels. The qualitative component of data analysis was then conducted by two parallel panels of subject matter experts (SMEs), one panel consisting of the four coders and another panel consisting of four non-coder SMEs with extensive knowledge of MTS research. All analysts underwent training during which they were briefed on the study design and best practices for qualitative data analysis and theme development (Ryan and Bernard, 2003; Braun and Clarke, 2006). Each panel separately engaged in detailed analysis of a subset of 24 cases; approximately 20% of cases were analyzed by both panels. Each panel used the MTS mappings, timelines, raw codes, and quantitative summaries of each case to inform an in-depth discussion of the similarities across the cases, including patterns of behaviors and processes observed and their likely explanations based upon current theory. Following separate discussions, the coder and non-coder panels came together to compare and synthesize the uncovered patterns into a list of coherent themes and related explanatory mechanisms pertaining to the research questions.

## Findings

The analyses produced findings comprising four overarching themes. The first two themes respond to the first research question, while the third and fourth themes respond to the second research question. First, we present a brief, overall summary of the data.

### Summary of the Data Set

The data set comprises 40 cases of failed MTS performance from six different organizational contexts, including business, government, emergency response (including both firefighter-only and large-scale, cross-organizational response efforts, such as those including police, fire, medical, and special response teams), science, military, and transportation (including flight and railway); see Table 2 for a full list of all cases. A total of 3,203 behaviors were identified; see Table 3 for a summary. All behaviors from the taxonomy were coded in at least one of the cases and no less than 4 times (see the Max column of Table 3). However, every behavior was also *not* represented in at least one case (see the Min column of Table 3). This has positive implications for the generalizable utility of the MTS action subphase taxonomy as a diagnostic tool in MTS research and practice (see our discussion of this point in the Applications section).

**Table 3:** Frequencies of Occurred Codes across the Cases.

Level	Subphase	Behavior	Freq	Percent (%)	Avg	Med	Min	Max
Within	Acting^*^	1	517	16.14	12.83	8	0	54
Within	Acting^*^	2	217	6.77	5.30	3	0	35
Within	Acting^**^	3	14	0.44	0.36	0	0	6
Within	Acting^**^	4	178	5.56	4.48	2	0	55
Within	Acting^**^	5	101	3.15	2.53	1	0	37
Within	Monitoring^+^	6	54	1.69	1.35	0	0	19
Within	Monitoring^+^	7	34	1.06	0.91	0	0	10
Within	Monitoring^+^	8	142	4.43	3.56	1	0	34
Within	Monitoring^++^	9	94	2.93	2.33	0	0	52
Within	Monitoring^++^	10	53	1.65	1.33	0	0	21
Within	Monitoring^+^	11	205	6.40	5.13	3	0	26
Within	Monitoring^++^	12	51	1.59	1.28	0	0	13
Within	Recalibrating	13	90	2.81	2.31	0	0	64
Within	Recalibrating	14	113	3.53	2.81	0	0	34
Within	Recalibrating	15	146	4.56	3.63	1	0	29
Between	Acting^*^	16	298	9.30	7.33	5	0	26
Between	Acting^*^	17	21	0.66	0.58	0	0	6
Between	Acting^*^ [Table-fn tfn1]	18	9	0.28	0.23	0	0	4
Between	Acting^**^	19	38	1.19	0.91	0	0	11
Between	Acting^**^	20	35	1.09	0.90	0	0	13
Between	Monitoring^+^	21	142	4.43	4.03	2	0	33
Between	Monitoring^++^	22	75	2.34	1.80	1	0	12
Between	Monitoring^++^	23	110	3.43	2.75	2	0	19
Between	Monitoring^++^	24	37	1.16	0.93	0	0	15
Between	Monitoring^+^	25	61	1.90	1.48	1	0	14
Between	Monitoring^++^	26	114	3.56	2.81	2	0	18
Between	Recalibrating	27	50	1.56	1.23	0	0	17
Between	Recalibrating	28	71	2.22	1.73	0	0	14
Between	Recalibrating	29	133	4.15	3.31	1	0	25

Freq represents the total frequency of the coded behaviors across all cases. Percent represents the percentage of the given behavior of all coded behaviors across all cases. Avg, Med, Min, and Max represent the average, median, minimum, and maximum number of times the given behavior was coded per case. The gradient green shading in the Frq and Percent columns represents the proportion of a given cell across the column from lowest (white background) to highest (darkest green background).

* Aligning-type acting subphase behaviors;

** Adjusting-type acting subphase behaviors.

+ Tracking-type monitoring subphase behaviors;

++ Communicating-type monitoring subphase behaviors.

### Theme #1: The Component Teams of Failing MTSs Over-Engage in Within-Team Alignment Behaviors (vs. Between-Team Behaviors)

Answering first research question, we observed that, across all cases of failed MTS performance, component teams demonstrated more within-team alignment behaviors than between-team behaviors, shown by the total number of behaviors coded at each level. Specifically, we coded 66% within-team behaviors and 33% between-team behaviors. Overall, members of these failing systems tended to engage in behaviors within their given teams to facilitate between-team alignment more often than they engaged in between-team interactions. This general pattern held at the subphase level such that acting, monitoring, and recalibrating behaviors each occurred more often within than between teams. In other words, team members were more likely to enact goal-striving behaviors within their own team, monitor their own team, and plan for adjustment with their own team members than they were to engage any such behaviors with members of other teams. Additionally, when acting and monitoring behaviors were examined within each of their behavior types, aligning, adjusting, and tracking behaviors all occurred more often within teams than between teams. As the sole exception, communicating behaviors occurred more between teams than within teams, a point we discuss below. See Figure 1 for a visual depiction of the proportion of within- vs. between-team behaviors overall, at the subphase level, and at the subphase behavior type level.

**Figure 2:**
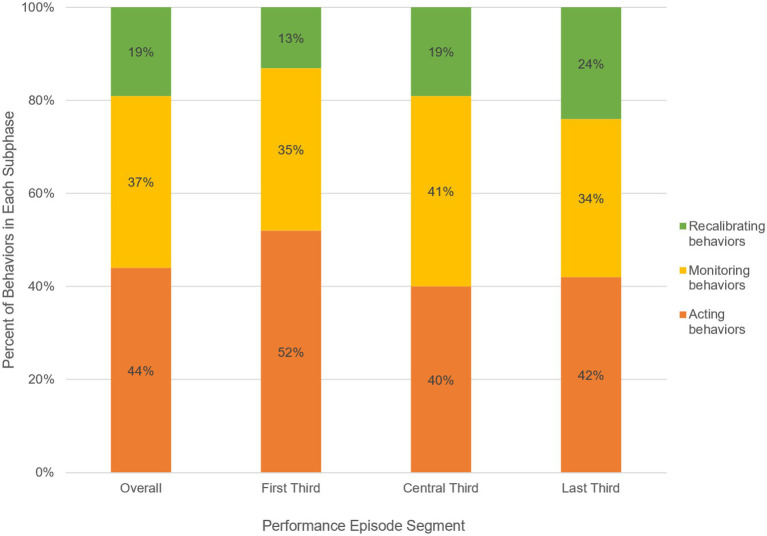
Percent Behaviors in Each Subphase Overall and over the Action Phase of the Performance Episode. The y-axis represents the percentage of each behavior that was coded within each subphase of action performance. The x-axis includes a column for the coded behaviors that occurred both overall and within each section of the performance episode. For example, the furthest right column indicates that, of all of the behaviors coded in the last third of the performance episode, 42% were acting, 34% were monitoring, and 24% were recalibrating.

**Figure 3:**
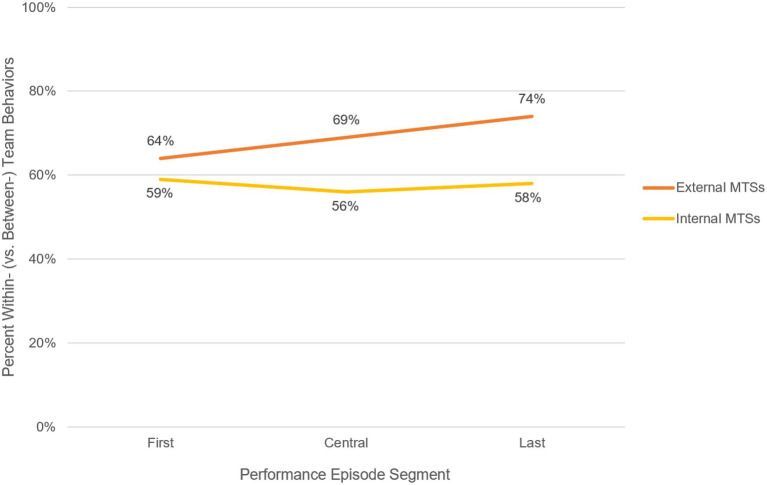
Change in Within vs. Between Behaviors over the Action Phase of the Performance Episode by MTS Boundary Status. The y-axis represents the percentage of within-team behaviors, relative to the percentage of between-team behaviors. The x-axis indicates the coded behaviors that occurred within each section of the performance episode. For example, the furthest right data point on the lower line indicates that, for all coded behaviors in the last third of the performance episode, for cases of performance for MTSs with internal boundary status, the behaviors were 58% within-team.

Further, when examined across the action phase of the performance episodes, the pattern of more within behaviors increases slightly. That is, across all cases, component teams’ behaviors increasingly occur more often within than between teams (see Figures 2, 3). This suggests a dynamic aspect to this trend such that, not only are teams constantly focusing their behaviors and interactions internally, but they increase this inward focus as their situation persists.

This first overarching theme echoes other research on collective collaboration. Past MTS research has indicated that component teams, and especially failing teams and teams under stress, turn their attention and behaviors inwards (Kanfer and Kerry, 2012; Beck and Plowman, 2014; Stoate, 2015; Wijnmaalen, 2015; Rico et al., 2016, 2018; Bick et al., 2017; Schecter, 2017; Schipper, 2017; Schipper and Gerrits, 2017; Wijnmaalen et al., 2018, 2019; Pendergraft et al., 2019; Ziegert et al., 2020; Torres et al., 2021). Both cognitive and motivational mechanisms explicate this trend in failing MTSs. Even under typical circumstances, research suggests that component teams may interact more within themselves than between or among themselves. Kanfer and Kerry (2012) suggest that this may be because within-team interactions represent a strong situation with established norms, while between-team interactions represent a weaker situation.

Another perspective suggests that stressful circumstances magnify social identity processes (e.g., Klein et al., 2006). Because teams experience an increased cognitive load inherent when responding to atypical circumstances, they may favor cognitively less-taxing interactions within their teams rather than engaging the increased effort associated with between-team interactions. This suggests that triggers, including various types of stress and crises, act as boundary-enhancing forces, which serve to “maintain component team distinctiveness even as they work together on integrated goals” (DeChurch and Zaccaro, 2013, p. 17). As such, Rink et al. (2013) suggest that teams under stress turn inward for the sake of familiarity, which engenders trust and commitment and may facilitate effective and increased coordination over time. However, as teams stay more focused on within-team alignment and engage in more within-team behaviors, they are more likely to lose sight of between-team obligations and behaviors imperative to reaching the system’s superordinate goal. Thus, such triggers pull the component teams’ focus and engagement inward, within the team, rather than outward and upward to the system level. Allison and Shuffler (2014) found a similar effect in their case study analysis of a financial services industry MTS facing challenges associated with an organizational change effort.

Our first theme suggests that a contributing factor for these MTSs’ failures was their inordinate attention to within-team interactions at the expense of between-team processes. This aligns closely with Torres and colleagues’ finding that the three 2017 collisions of Navy destroyers were largely influenced by insufficient between-team behaviors, which they based upon the behavioral-level comparison of behaviors enacted during each of the failed cases with those recommended for optimal performance by Navy SMEs. In the failing MTS cases in our dataset, the tendency to turn inward also seemed to be counter-effective for achieving the systems’ superordinate goals. Instead, because component teams seemed to be disincentivized from the less-familiar interactions between each other, they were less likely to sufficiently collaborate and coordinate, which in turn exacerbated the overall ineffectiveness of their responses.

For example, the flight team of DANACO flight 992 briefly considered calling upon the Engineer on the Lagos Area Control Centre team for assistance in solving the sudden, unexplained non-response of the left engine to throttle movement; however, they immediately dismissed this idea, with the Captain saying “Well, I do not need him here, ‘cause we can figure it out. He’s not going to be able to help us.” (Accident Investigation Bureau of Nigeria, n.d., p. 71). The flight crew failed to interact with any other teams in the system until nearly 30 min later, when they contacted Area Control to declare an emergency due to the situation’s continual worsening. Approximately 2 min after the emergency declaration, the airplane crashed, resulting in the fatalities of all 153 people on board. This situation demonstrated a failure of the team to engage in sufficient and timely between-team interaction.

To check the assertion that a greater (or at least equal) volume of between-team interactions, compared to within-team interactions, are critical for successful MTS performance across additional contexts, we examined a standard operating protocol (SOP) for flight team and air traffic control (ATC) interactions which prescribes the ideal within- and between-team behaviors that should occur during the landing phase of performance. As cases of successful MTS performance are largely undocumented and/or unavailable, these protocols served to represent cases of MTS success to contrast against the failure cases. That is, MTSs that follow such protocols should achieve successful performance. As with the failed MTS performance cases, we used the MTS action subphase taxonomy to code the behaviors throughout the SOP, and, overall, we found a higher proportion of between-team (69%) than within-team (31%) behaviors. This presents a stark contrast against the most similar cases of failed performance for flight-ATC MTSs in our dataset, which demonstrated only 16% between-team behaviors (vs. 84% within) and supports our foundational assertion that between-team behaviors are critical for MTS successful performance.

In addition to offering support for the notion that failing systems and those under stress too frequently turn their attention and behaviors inwards, we provide nuance describing just how pervasive the trend to over-rely on within-team interaction may be across different types of MTSs. Teams embedded in MTSs seemed to engage in nearly all types of behaviors more so at the within- (vs. between-) team level, and this tendency only increased as the situation persisted. This trend may imply that the coordination between teams happens more implicitly than explicitly, as would be required for effective between-team collaboration (Rico et al., 2008, 2019). Notably, however, the occurrence of more between- than within-team communication monitoring behaviors did not offer a sufficient counter. Though the component teams of these failing MTSs seemed to be attempting explicit coordination in light of their situations, their communication was largely focused on system-level goal progress, the appearance of potential triggers, and alerting other teams when triggers were realized (see Table 2 behaviors # 22, 23, 24, and 26). These communications were not those that facilitate a shared understanding of the situation, the nature of the trigger, and the appropriate response (i.e., recalibrating and behavior #27). As such, the frequency of between-team monitoring-communicating behaviors may be an insufficient counter for dearth of between-team recalibrating. Teams’ between-team communications seemed insufficient to facilitate adequate adjustment and ensure the ultimate success of the MTS in achieving its superordinate goal.

### Theme #2: Failing MTSs Do Not Fully Enact the Action Subphase Cycle

**Figure 4:**
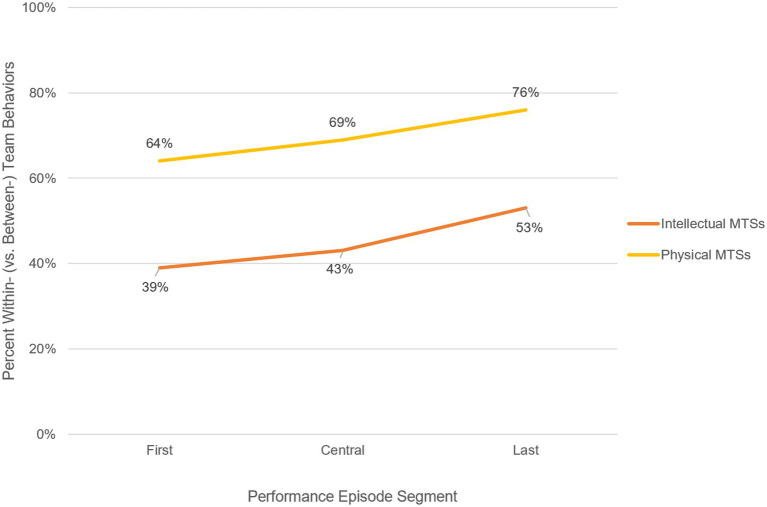
Change in Within vs. Between Behaviors over the Action Phase of the Performance Episode by MTS Goal Type. This descriptive evidence presented in this figure and throughout this theme is based upon only cases of external physical MTSs (*n* = 25), as the data set only includes cases of external intellectual MTSs (*n* = 5) due to limited availability and accessibility of cases of internal MTSs; this is discussed further in the Limitations section. By removing the cases of internal physical MTSs (*n* = 10) from this comparison, we aimed to mitigate confounding the effects of boundary status with those of goal type. The y-axis represents the percentage of within-team behaviors, relative to the percentage of between-team behaviors. The x-axis indicates the coded behaviors that occurred within each section of the performance episode. For example, the furthest right data point on the lower line indicates that, for all coded behaviors in the last third of the performance episode, for cases of performance for MTSs with intellectual goal types, the behaviors were 53% within-team.

In the first theme, we describe teams’ over-reliance on within-team alignment behaviors compared to between-team behaviors that are critical for successful MTS performance. The second theme emerged from consideration of the relative prevalence of behaviors at the subphase level. Of the three subphases of MTS action (i.e., acting, monitoring, and recalibrating), across all cases of failed MTS performance, component teams engaged most often in acting behaviors, less in monitoring, and least in recalibrating (see Figure 4, Overall column). That is, the teams were more often engaged in goal-striving behaviors than in tracking, communicating, or developing plans for adjustment and/or adaptation. Though recalibrating behaviors were the least frequently observed, trends show that component teams increasingly enacted them over the course of the action phase. That is, teams generally made more efforts to adjust and adapt as the action phase persisted; however, as all cases represented failing MTSs, these efforts were likely too little and/or too late.

Additionally, the findings show that, when the failing MTSs engaged in acting, it was mostly aligning (74% of all acting behaviors, vs. 26% adjusting); however, like recalibrating, component teams engaged in increasingly more adjusting over the course of the performance episode, from 12% in the first third, to 29% in the central third, to 40% in the last third. This again suggests that the systems were attempting to respond to the triggers, challenges, and stressors they faced, but did so insufficiently. Finally, patterns showed that, when failing MTSs engaged in monitoring, it was mostly tracking (54% of all monitoring behaviors, vs. 46% communicating). This trend increased slightly over the course of the action phases, from 52% (vs. 48% communicating) in the first third, to 54% in the central third, to 57% in the final third. That is, when teams should have been engaging in more communications to monitor the effectiveness of their behaviors, including adjustment behaviors, they instead engaged in the more passive tracking behaviors indicative of a “wait-and-see” approach. See Figure 4 for a visual depiction of the proportion of acting, monitoring, and recalibrating behaviors overall and across each section of the performance episode.

Taken together, these patterns suggest that the component teams of the failing MTSs were insufficiently responding to the crises they faced. Though they showed slight increases in recalibrating and adjusting behaviors over the course of the action phase, these shifts were likely too slow to afford effective response. Regardless of their efforts, these MTSs still failed to achieve their superordinate goals. Additionally, decreasing communicating behaviors over the performance episode may have further inhibited their ability to adequately engage in effective recalibration and adjustment, particularly between teams as described in the first theme.

For example, the response effort to the 2018 grass fire in Texas presents a dramatic example where early mistakes went unchecked throughout the action phase of performance. In this case, a large system of over 10 component teams responded to an in-progress grass fire. For undetermined reasons, two firefighters from one team failed to wear proper personal protective equipment for the duration of the performance episode, with no corrective action taken by any of the other responding personnel, which resulted in life-threatening injuries for one firefighter and fatal injuries for the other. Our findings suggest that, because the other teams were so focused on their own acting behaviors, they failed to engage sufficient monitoring or recalibrating behaviors to catch the error and prevent the subsequent injuries (National Institute of Occupational Safety and Health Fire Fighter Fatality Investigation and Prevention Program, 2019). This example demonstrates cross-level impact wherein within-team alignment and between-team behaviors, over time, impact system performance. In this case, within-team actions impacted between-team actions and prevented the system’s safe and timely extinguishing of the grass fire.

Compared to the behavioral trends represented in these cases, successful MTSs response to crisis situations would be expected include more recalibrating, adjusting, and communicating to facilitate a coordinated and collaborative response. This is proposed by Torres et al.’ (2021) action subphase model wherein, through an iterative behavioral cycle, MTS components teams, under the atypical and responsive circumstances that follow the acknowledgement of a trigger *via* tracking monitoring behaviors, move on to communicating monitoring behaviors, and then to recalibrating, and finally to adjusting acting. However, this larger, *adjustment-focused cycle* is predicated by a smaller, *alignment-focused cycle*, during which component teams, under typical and familiar circumstances, enact recurring iterations of aligning acting and tracking monitoring behaviors. It is possible that teams that spend far more time in the alignment-focused cycle become entrained to it.

Entrainment is a phenomenon of synchronized alignment of behavioral cycles (Ancona, 1990; Ancona and Caldwell, 1988, 1992; Ancona and Chong, 1996) or the enactment of “interconnected rhythmic patterns of activity” by component teams in MTSs (Standifer, 2012, p. 396). Component teams’ tendency to entrain the alignment-focused cycle may be exacerbated by a manifested complacency that drives teams to continually return their most frequently performed tasks (Luciano et al., 2018a). As such, for these failing MTSs, there seemed to be over-reliance on the familiar that continually returned them to the alignment-focused cycle, when they should have instead shifted to the adjustment-focused cycle. Whether the teams failed to acknowledge the trigger or to communicate the need for adjustment or adaptation, which would have pushed the system forward into recalibration, all of these failed cases showed a lack of timely or fully engaging the adaptation cycle.

### Theme #3: Boundary Status Exacerbates Failing MTSs’ Component Teams’ Tendency to Engage in Insufficient Between-Team Behaviors

Regarding the second research question, we observed that the patterns described in the first and second themes were found to be exacerbated by MTS boundary status. External MTSs, composed of component teams from different organizations who often have higher differentiation (i.e., “the degree of difference and separation between MTS component teams at a particular point in time,” Luciano et al., 2018b, p. 1067), more strongly demonstrated (1) the tendency to over-engage in within- (vs. between-) team behaviors and (2) the failure to enact the full, adjustment-focused action subphase cycle. Internal MTSs also displayed these behavioral patterns, which likely contributed to their performance failures, but the trends were weaker compared to external MTSs.

The differing strength of each MTS type became even more apparent when examined across the action phase (see Figure 2). While external MTSs increasingly engaged in more within-team behaviors, the proportion of within (versus between-team behaviors) for internal MTSs decreased very slightly before remaining relatively stable through the performance episode. That is, the component teams in external MTSs turned more and more inward as their situation persisted, while those in internal MTSs demonstrated far less change over time in how engaged they were within versus between teams.

Additionally, the proportions of communicating versus tracking monitoring behaviors showed opposite trends for internal and external MTSs. Specifically, while the proportion of tracking decreased and leveled off for internal MTSs (65% tracking vs. 35% communicating in the first third to 53% tracking vs. 47% communicating in the central and last thirds), it steadily increased for external MTSs (from 45, to 55%, to 60% tracking vs. communicating). External MTSs seemed increasingly resistant to enacting communicating behaviors, especially between teams, as the action phase persisted. While the shifts in percentages are relatively small changes across all cases in aggregate, the differing patterns that emerged suggests that failure manifests differently for these different types of MTSs, thus providing theoretical nuance to enlighten failure processes in MTSs. See Figure 2 for a visual depiction of the shifting proportion of within- vs. between-team behaviors across each section of the performance episode for MTSs with external vs. internal boundary statuses.

Taken together, these ideas suggest that component teams in external MTSs not only face additional barriers due to differentiation (Luciano et al., 2018b), but that these barriers most strongly affected behaviors that are less directly goal striving and not a part of their most typical and familiar behavioral cycle. While the behavioral patterns demonstrated by failing internal and external MTSs are not wholly different, they appear to be stronger for MTSs with component teams from different organizations (i.e., external MTSs). Though it is certainly not impossible for internal MTSs to fail and to do so with catastrophic results, the reduced challenges faced by such systems, particularly those with low differentiation, may lessen the likelihood of system-level failure and/or mitigate its impact, relative to external MTSs.

To demonstrate, different patterns emerged across the Naval ship collisions. The collision of USS *Essex* (LHD 2) with USNS *Yukon* (T-AO 202), an internal MTS given the ships’ shared organizational membership in the United States Navy, showed more within-team than between-team behaviors overall, but in steady proportion across the action phase. Most between-team behaviors were tracking monitoring behaviors (e.g., “YUK Master observing ESX bow coming starboard”; Department of the Navy, 2012, p. 13). Comparatively, the collision between USS *Frank E. Evans* (DD 754) with HMAS *Melbourne* (R 21), an external MTS given the ships’ differing organizational membership with the U. S. Navy and the Royal Australian Navy, respectively, showed steadily more within-team behaviors over the course of the action phase. The between-team behaviors that occurred were largely communication, including the notable final transmission from the *Evans* Deck Officer to the *Melbourne*: “Roger, my rudder is right full over” (Judge Advocate General, 1969, p. 16) that occurred seconds before the collision point that resulted in the deaths of 74 sailors. These cases, when examined in parallel, highlight differences in how failed performance is enacted that may be attributed to the forces in play based on MTS boundary status.

### Theme #4: Goal-Type Exacerbates Failing MTSs’ Component Teams’ Tendency to Engage in Insufficient Between-Team Behaviors

MTS goal type also appears to moderate the behavioral trends described in the first and second themes. Specifically, physical MTSs seem much more subjected to the tendency to overly engage in within-team action processes. Overall, physical MTSs engaged in more within-team behaviors (70 vs. 30% between-team), while intellectual MTSs engaged in slightly more between-team behaviors (45 vs. 55% within-team).

When considered over the course of the action phase, both physical and intellectual MTSs show a steady increase of within-team behaviors (see Figure 3). However, intellectual MTSs, even at their highest proportion of within-team behaviors (53% in the last third), demonstrate fewer within-team behaviors than physical MTSs at their lowest (67% in the first third). This same pattern occurred at the subphase level for both types of MTSs; that is, acting, monitoring, and recalibrating behaviors all occurred more within teams for physical MTSs and more between teams for intellectual MTSs. Notably, this pattern of different behavioral levels (i.e., within vs. between-team) across physical and intellectual MTSs was not replicated in monitoring behavior type (i.e., tracking vs. communicating). Communicating behaviors seemed to be no more strongly affected than other behavior types, as both physical and intellectual MTSs showed a steady decrease in communicating monitoring behaviors (in favor of tracking monitoring behaviors) over the action phase. See Figure 3 for a visual depiction of the shifting proportion of within- vs. between-team behaviors across each section of the performance episode for MTSs with intellectual vs. physical goal types.

Taken together, these ideas suggest that component teams in physical MTSs, as compared to those in intellectual MTSs, face different and perhaps stronger barriers to collaboration, inhibiting between-team behaviors but not adaptive behaviors overall, that may be tied to the nature of their goal. These barriers may be further inhibited by differing leadership structures of external and internal MTSs, as discussed in the Contributions section.

These differences are apparent when comparing two physical and intellectual goal-type MTS cases of failed performance. In the 2013 Navy Yard active shooter response case, an MTS with a goal rooted in physical execution, the component teams engaged in 58% within-team behaviors (vs. 42% between-team) across the course of the action phase. The ineffective between-team communications during the response effort were largely related to component teams from different organizations using different radio channels while on the scene, which hindered effective coordination and timely demobilization and increased risk to the responding personnel (Department of the Navy, 2013). Comparatively, in the 2013 Affordable Care Act website launch delay case of an MTS with an intellectual-type goal, the component teams engaged in 34% within-team behaviors (vs. 66% between-team) across the course of the action phase. In this case, communication issues began early in the action phase, with substantial delays in the needed website specifications from key stakeholders, that impacted the project’s later phases and ultimately resulted in its failure to meet the designated timeline (United States Government Accountability Office, 2014; Lee and Brumer, 2017). Taken together, these cases show that MTSs with different goal types can and do fail to achieve their superordinate goals, but that this may occur in different ways.

## Contributions of Understanding MTS Performance Failures: The Role of Entrainment

Throughout the findings, we argue that entrainment is a mechanism that facilitates MTS performance and that the behavioral cycles to which component teams are entrained function as a determinant of MTS failure. Other researchers have applied the concept of entrainment to small groups (Kelly and McGrath, 1990) and to MTS performance, most notably Standifer (2012). In this application, the specific, cyclical patterns of behavior enacted by component teams of MTSs align to facilitate goal achievement, like the interconnected gears of a watch, “moving at seemingly random paces… yet, by their coordinated, unified effort, one collective goal is reached—namely, you can tell the time” (Standifer, 2012, p. 395). Because the entrained behavioral cycles are interdependent, they directly influence MTS within- and between-team processes (Standifer, 2012). The behaviors enacted by component teams compose cycles, and the cycles that are entrained at any given time influence the cycles entrained by other teams. The degree to which these cycles are aligned between the teams and with the system’s overarching goal determines whether and how an MTS fails. That is, entrainment occurs through component teams’ cyclical enactment of within-team alignment and between-team behaviors and directly impacts MTS performance. Our study leverages this theoretical basis to uncover additional nuance and understanding of the mechanism and its boundary conditions.

**Figure 5:**
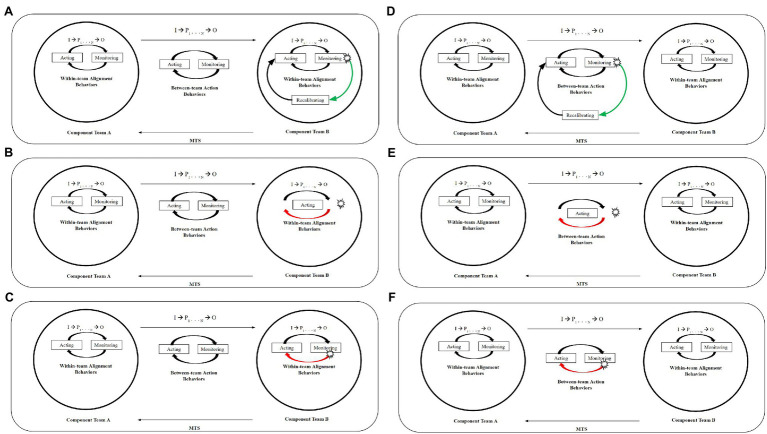
Expanded MTS Action Subphase Model for Successful and Misaligned MTS Responses to Within- and Between-Team Triggers. All figures are adapted from the original MTS Action Subphase model presented in Torres et al. (2021). Solid black rectangle represents the MTS boundary. Outlined circles represent component team boundaries. Straight arrows represent between-team interdependence. Trigger is indicated by blast shape. Curved arrows represent subphase relationships. Green curved arrow represents an appropriate response to the trigger. Red curved arrow represents an inappropriate response to the trigger. **(A)** Successful Adaptive MTS Response to Within-Team Trigger. The purpose of this figure is to visually depict a successful, subphase level response cycle to a within-team level trigger for adaptation. That is, this is what might occur if an MTS is operating under normal conditions, entraining the baseline behavioral cycle of acting (aligning-type behaviors) and monitoring (tracking-type behaviors) at the within- and between-team levels, when a team-level trigger occurs for Component B. In this instance, Component B shifts to a cycle that includes monitoring-communicating type behaviors, recalibrating behaviors, and acting-adjusting type behaviors. This cycle will continue until the trigger is resolved. The team may concurrently enact monitoring-tracking and acting-aligning type behaviors as well. As such, the figure above is depicted at the subphase level. **(B)** Misaligned Adaptive MTS Response to Within-Team Trigger *via* Acting-Focused Cycle. The purpose of this figure is to visually depict an unsuccessful, subphase level response cycle to a within-team level trigger for adaptation. That is, this is what might occur if an MTS is operating under normal conditions, entraining the baseline behavioral cycle of acting (aligning-type behaviors) and monitoring (tracking-type behaviors) at the within- and between-team levels, when a team-level trigger occurs for Component B. In this instance, Component B shifts to a cycle that includes only acting behaviors and, as such, is unlikely to resolve the trigger without the necessary monitoring and recalibration behaviors that are depicted in **(A)**. These acting behaviors may include alignment and/or adjustment types; therefore, the figure above is depicted at the subphase level. **(C)** Misaligned Adaptive MTS Response to Within-Team Trigger *via* Alignment-Focused Cycle. The purpose of this figure is to visually depict an unsuccessful, subphase level response cycle to a within-team level trigger for adaptation. That is, this is what might occur if an MTS is operating under normal conditions, entraining the baseline behavioral cycle of acting (aligning-type behaviors) and monitoring (tracking-type behaviors) at the within- and between-team levels, when a team-level trigger occurs for Component B. In this instance, Component B shifts to a cycle that includes only acting and monitoring behaviors and, as such, is unlikely to resolve the trigger without the necessary recalibration behaviors that are depicted in **(A)**. These acting and monitoring behaviors may include any combination of their behavioral types (i.e., acting-alignment and/or adjustment, monitoring-tracking, and/or -communicating); therefore, the figure above is depicted at the subphase level. **(D)** Successful Adaptive MTS Response to Between-Team Trigger. The purpose of this figure is to visually depict a successful, subphase level response cycle to a between-team level trigger for adaptation. That is, this is what might occur if an MTS is operating under normal conditions, entraining the baseline behavioral cycle of acting (aligning-type behaviors) and monitoring (tracking-type behaviors) at the within-team level, when a between-level trigger occurs. In this instance, the between-team behavioral cycle shifts to include monitoring–communicating type behaviors, recalibrating behaviors, and acting-adjusting type behaviors. This cycle will continue until the trigger is resolved. The teams may concurrently enact between-team level monitoring-tracking and acting-aligning type behaviors as well. As such, the figure above is depicted at the subphase level. **(E)** Misaligned Adaptive MTS Response to Between-Team Trigger *via* Acting-Focused Cycle. The purpose of this figure is to visually depict a successful, subphase level response cycle to a between-team level trigger for adaptation. That is, this is what might occur if an MTS is operating under normal conditions, entraining the baseline behavioral cycle of acting (aligning-type behaviors) and monitoring (tracking-type behaviors) at the within-team level, when a between-level trigger occurs. In this instance, the between-team behavioral cycle shifts to include only acting behaviors and, as such, is unlikely to resolve the trigger without the necessary monitoring and recalibration behaviors that are depicted in **(D)**. These acting behaviors may include alignment and/or adjustment types; therefore, the figure above is depicted at the subphase level. **(F)** Misaligned Adaptive MTS Response to Between-Team Trigger *via* Alignment-Focused Cycle. The purpose of this figure is to visually depict a successful, subphase level response cycle to a between-team level trigger for adaptation. That is, this is what might occur if an MTS is operating under normal conditions, entraining the baseline behavioral cycle of acting (aligning-type behaviors) and monitoring (tracking-type behaviors) at the within-team level, when a between-level trigger occurs. In this instance, the between-team behavioral cycle shifts to include only acting and monitoring behaviors and, as such, is unlikely to resolve the trigger without the necessary recalibration behaviors that are depicted in **(D)**. These acting and monitoring behaviors may include any combination of their behavioral types (i.e., acting-alignment and/or adjustment, monitoring-tracking, and/or -communicating); therefore, the figure above is depicted at the subphase level.

For MTSs in the action phase of performance, Torres et al. (2021) describe the ideal cycle that MTSs should entrain to successfully respond to within- and between-team triggers for adjustment or adaptation. In Figure 5, we expand and adapt this model to depict a series of examples of how MTSs might successfully and unsuccessfully respond to such triggers. In successful responses, the identification of a trigger for adaptation, which occurs in the monitoring stage, initiates a realignment to an adjustment-focused behavioral cycle that includes recalibration. The shift to entrain the adjustment-focused cycle may occur at either the within-team level (see Figure 5A) or at the between-team level (see Figure 5D), dependent upon the level at which the trigger occurs. Notably, entraining a given cycle involves both attuning the changing requirements for goal achievement relative to a shifting environment and enacting the behaviors of the appropriate behavioral cycle. That is, successful entrainment involves consideration of shifting task requirements and task execution. In the exemplar cases of failed MTS performance described in the discussion of each theme above, this consideration and/or execution are missing from the systems’ responses to the situations. Though not specific to MTS performance, early work on entrainment by Ancona and colleagues suggests that trade-offs between such requirements may be key determinants to team performance (Ancona, 1990; Ancona and Caldwell, 1988, 1992).

In unsuccessful responses to triggers, however, the teams and system fail to entrain the adjustment-focused cycle described above. Instead, in the cases in the current study, teams may make two possible entrainment-focused errors. First, they fail to make any change or adjustment, instead remaining entrained to the alignment-focused cycle that they were in before the trigger occurred (see Figure 5B for this failed response to a within-team trigger and Figure 5E for this failed response to a between-team trigger). This may happen when a team chooses to continue to monitor a threat, underestimating its potential impact, or when they are unaware of the threat entirely. As this cycle is only appropriate for typical performance conditions, remaining entrained to it, or returning to it too soon or too frequently, rather than fully entraining the adjustment-focused cycle, may result in an insufficient response to the trigger and, ultimately, failed performance.

A second, and perhaps worse still, unsuccessful response occurs when teams shift and entrain a different, but smaller behavioral cycle that includes only acting behaviors (see Figure 5C for this failed response to a within-team trigger and Figure 5F for this failed response to a between-team trigger). This acting-focus cycle frequently appeared in the data when component teams were unaware of a specific trigger, but recognized that their performance was decreasing. For example, recalling the previous example of the flight team, they may have noticed warning signals but, rather than engaging the air traffic control team, continued to try to address the problem themselves by adjusting the plane’s speed or wing flap position. Thus, in these instances, rather than increase their monitoring, they engaged various responsive actions repeatedly until the system failed to reach its overarching goal. In this way, both the alignment- and acting-focused cycles are considered inappropriate for situations that call for adaptive responses to triggers which MTSs frequently face.

Notably, the ideal behavioral responses to triggers proposed in Torres et al.’s (2021) taxonomy are multilevel, indicating that effective entrainment of the adjustment-focused cycle includes within- and between-team behaviors to ensure success. As described in theme 2, component teams of MTSs tend to entrain their most familiar behavioral patterns (i.e., within-team behaviors and the alignment-focused cycle) in efforts to preserve their cognitive effort (Kanfer and Kerry, 2012). However, returning to the watch metaphor, this tendency can be problematic because, when a gear in the system is on a different cycle or pace than those with which it is interconnected, the whole system locks up and shuts down. For MTSs, component teams’ over-reliance on within-team alignment behaviors may either lead to or become a function of their loss of focus on the system’s superordinate goal. That is, when component teams are overly focused on their proximal goals, they in turn decrease their focus on the MTSs’ distal goals (Allison and Shuffler, 2014). This tendency can be particularly prevalent and especially harmful when the system’s goal is under threat. As such, component teams’ untimely entrainment to team-level behavioral cycles may inhibit their capacity to coordinate between teams and achieve goals that rely on interdependent collaboration (i.e., the MTS superordinate goal).

The tendency to too frequently entrain within-team behavioral and/or and alignment-focused cycle may be a function of inherent incompatibilities within the MTS goal hierarchy. MTS theory suggests that goal compatibility is not automatic in MTS contexts (Davison et al., 2012; Rico et al., 2016). What is most beneficial for the component teams and their proximal goals is not necessarily most beneficial for the MTS and its superordinate goal (DeChurch and Zaccaro, 2013). It is possible that within-team focus, particularly during stressful circumstances, may be the result of a conscious, “not-my-job” redirection of focus wherein team members’ choose to direct their attention and efforts toward the closest (i.e., proximal) goals for their more salient identification with their component team. This shift might be motivated positively (i.e., trusting the other component teams to do their part) or negatively (i.e., determinedly pursuing the proximal, team-level goal even at the cost of the system goal). Regardless of its motivations, this tendency to pull inward, both effort and attention, within the team seems to exacerbate failing performance at the MTS level. When teams most need to coordinate across their boundaries to address an evolving threat to goal achievement (i.e., the MTS superordinate goal), they reroute their efforts instead to maintaining their most salient goals and most familiar tasks.

### The Interaction Between Entrainment and MTS Attributes

#### MTS Boundary Status

The failure to engage in much-needed boundary crossing for collaborative response may be exacerbated by an MTS’s boundary status. Members of external MTSs must overcome the stronger and often more salient between-team boundaries, compared to the boundaries between teams of internal MTSs. Because of this, members of external MTSs seem to be even less likely than internal MTS members to cross between-team boundaries. It is important to note that, though the failing patterns as described in the themes above seem weaker for internal MTSs, this does not imply that they cannot fail or that they fail less severely than external MTSs. It does suggest, however, that failing external MTSs may be subjected to additional barriers inhibiting their capacity to engage in sufficient and timely adjustment and adaptation.

Boundary-enhancing forces between component teams may be augmented by the increased MTS differentiation (Luciano et al., 2018b) in external MTSs, and it may be particularly difficult for teams to effectively manage different and potentially even competing identities with their team, system, and organization to effectively coordinate and collaborate. Instead, they seemed to engage in less taxing, more familiar within-team interactions, perhaps at the expense of the MTS superordinate goal. For external MTSs, this effect seemed stronger because boundaries between the teams are inherently stronger—teams needed to expend both social and cognitive (DeChurch and Zaccaro, 2013) effort to overcome both team- and organizational-level identities. This finding echoes recent teams literature highlighting the difficulty for teams to overcome “thick” boundaries, including those pertaining to spheres of knowledge, particularly when engaged in interdependent problem-solving tasks (Kerrissey et al., 2021).

Another factor strengthening the barriers between MTSs of different boundary statuses may be differing leadership structures. Internal MTSs, with all component teams represented by a single organization, likely have a more hierarchical leadership structure, while external MTSs may have a more distributed leadership structure that is shared among leaders from each organization represented in the system. Because leaders drive the MTS team behaviors (DeChurch and Marks, 2006), they also help facilitate the behavioral cycles entrained by the teams and system as a whole. For systems with multiple leaders, a lack of shared awareness among leaders can cause confusion and misalignment among component teams, and this may be more likely to affect MTSs wherein the leaders represent different organizations (i.e., external MTSs) with different roles, functions, proximal goals, and levels of responsibility. Though internal MTSs may also have multiple leaders or be subject to the negative impact of misaligned situational awareness, external MTSs may be more subjected to this threat. In a recent multimethod study of Dutch railway MTSs, de Vries et al. (2021) suggest that component teams from external MTSs initially experience parochial viewpoints and interests or “a narrow focus on home team activities and interests and a lack of awareness of and concern for the interests and activities of other teams” (p. 7). However, through leader-driven boundary management efforts over time, these systems may overcome such boundary-enhancing forces and shift to integrated pluralism, or a state of “richer and more frequent communication while simultaneously respecting and defending each team’s distinct operational environment and home organization” (de Vries et al., 2021, p. 9), which ultimately serve to improve the MTSs’ performance.

#### MTS Goal Type

Like boundary status, MTS goal type may exacerbate a system’s failure to engage in necessary boundary crossing for goal achievement. These trends may be explained through contrasting the core nature of physical vs. intellectual MTSs. Intellectual MTSs exist primarily to facilitate collaboration and often innovation among the component teams (Zaccaro et al., 2012). This guiding objective may allow component teams to override the tendency to focus within, particularly in times of stress or crisis, and instead lean into the most salient goal (i.e., collaboration) and engage behaviors that most directly facilitate this goal (i.e., between teams). Intellectual MTSs may be just as prone to entraining the alignment-focused cycle over the adjustment-focused cycle; however, the action behaviors of their alignment-focused cycle (i.e., communication and coordination) may more readily allow a shift to entraining the adjustment-focused cycle that facilitates collaborative response to triggers for adaptation. The action behaviors of physical MTSs, while perhaps requiring communication for successful collaboration, may be less readily tied to communication (e.g., suppressing fire, operating a plane). That is, while physical MTSs collaborate as a means to achieve some other goal, for intellectual MTSs, the collaboration is often the end itself.

Another factor driving the differences between MTSs with different goal types may be the inherently different rhythms of the alignment-focused cycles of intellectual and physical MTSs, evidenced by the extreme difference in the duration of the action phases of each type. The durations of intellectual MTS cases in this dataset range from approximately 2 years to approximately 10 years, while the physical MTSs cases range from 2 min to approximately 10 h. The extreme duration of the intellectual cases indicates an iterative performance rhythm wherein the component teams work in pendulum-like swings between longer stretches of separate, solo work interspersed with short bursts of intense collaboration (e.g., large cross-team meetings, conferences, or other project milestones). This punctuated equilibrium workflow has been described in teams literature (Gersick, 1988) and likely affects all types of MTSs; however, the performance episode duration and nature of the task may exacerbate this tendency in intellectual MTSs. As such, intellectual MTSs may experience a blurring of the bounds of the MTS itself, both in terms of its performance episode and as it relates to members’ perceived identity with their team and the MTS over time. Given the extended and punctuated workflow rhythm, intellectual MTSs members may be more likely to engage in multi-MTS membership, wherein members or even entire teams may have other projects that take their attention and, relatedly, their goal commitment (O’Leary et al., 2012). When intellectual MTSs do not succeed, it may be related to the failing MTS not being the primary focus for members or component teams outside of key MTS inflection points (Dubrow et al., 2017). Just as the tendency to overly focus within teams leads to less effort on the MTS goal and subsequently inhibits successful performance, so might intellectual MTSs’ entraining such extended cycles.

In sum, the findings of this study suggest that the phenomenon of entrainment serves as a plausible mechanism through which component teams of MTSs shift their behavior cycles in response to evolving circumstances. That is, MTS entrainment facilitates MTS adaptation and adjustment wherein the system re-aligns the interconnected behavior patterns of its component teams to achieve the system’s goals. When component teams fail to concurrently entrain the appropriate cycles, they may be more likely to fail to reach the MTS superordinate goal.

### Theoretical Implications

Though recent research has advanced understanding of the complex nature MTSs and their performance dynamics (e.g., Zaccaro et al., 2020), MTS researchers have recently called for empirical studies utilizing different methodologies with the aim of unifying different theories (e.g., Mathieu et al., 2018; Rico et al., 2018; Shuffler and Carter, 2018). Our findings expand and add nuance to current research on MTS performance, particularly regarding how MTS failure occurs and manifests differently for different types of MTSs, through the application of historiometric analysis methods. Additionally, the particular data set and methodology employed offer augmented generalizability to previously recognized trends. Though single-context studies of various types of MTSs have shown the importance of between-team interactions and the tendency for failing systems to over-engage within component teams, we demonstrate this finding in a cross-context comparison of failure cases. We also elaborated upon the role of entrainment as it relates to extant theory on the MTS performance (Standifer, 2012) with the aim of explicating underlying mechanisms that may drive success and failure in these high-stakes scenarios.

Literature to date generally supports the importance of between-team behaviors for effective coordination and collaboration and the potential threat to the goal of component teams’ foregoing these behaviors. MTS research also suggests that teams may lose sight of the system’s superordinate goal and restrain their efforts to within-team behaviors, subsequently inhibiting goal achievement (e.g., Allison and Shuffler, 2014). Though the trends suggested by this first theme are largely reflected in current MTS research, the present study offers nuance regarding when these patterns occur and how they affect MTS performance outcomes. First, while single-context case studies have separately found this trend, our comparative approach produced findings representing MTSs in various contexts and with differing attribute profiles that all seem to demonstrate the tendency to over-engage in within-team alignment behaviors compared to between-team behaviors.

Additionally, our use of Torres et al.’s (2021) action subphase behavioral taxonomy allowed the specification of the types of behaviors that are most likely subjected to this effect. That is, all acting, monitoring, and recalibrating behaviors (except, as noted above, communicating behaviors). The taxonomy also allowed for narrowed focus on within-team alignment behaviors—that is, those behaviors conducted within teams that facilitate between-team coordination and system-level goal accomplishment. Other studies (e.g., Marks et al., 2005; Davison et al., 2012) have not explicitly described team-level behaviors and distinguished those that simply serve the team’s internal functioning from those that specifically facilitate between-team effectiveness. Finally, this study comprises a temporal examination of system behaviors that has long been called for in MTS research (e.g., Mathieu et al., 2018; Zaccaro et al., 2020). Specifically, our methodological approach allowed for support to emerge for the continuance and strengthening of the observed patterns over time. We aimed to explore a possible mechanism (i.e., entrainment) that may explain component teams’ tendency to over-focus within themselves and how MTS’s attributes (i.e., boundary status and goal type) may impact this tendency. As such, we hope to provide both deeper and broader insight to how and why MTS performance failures occur.

### Practical Applications

This study offers support for the content validity of Torres et al.’s (2021) taxonomy of MTS action subphase behaviors, specifically in terms of its generalizable utility in efforts to capture and analyze MTS behaviors in practice. As noted in the Findings section, across the data set of considerably diverse cases of failed MTS performance, every behavior from the taxonomy was coded. This suggests that all behaviors from the taxonomy are enacted by varied types of MTSs at some point across the action phase of their performance episodes. That said, not all behaviors were coded in all cases, suggesting some variance in which behaviors are enacted by different types of MTSs and in different situations. As such, this suggests that the taxonomy may be utilized by practitioners to capture differing patterns of enacted behaviors by MTSs and their component teams across their performance episodes. The uncovered patterns indicate behaviors that are and are not enacted over time and may, therefore, be used for comparative evaluation of various MTS performance within and across systems and to answer questions related to practical aims, such as performance enhancement. As these are cases of failed MTS performance, however, practitioners and researchers should consider that, when using this taxonomy as a tool for performance assessment and diagnostics, the same patterns of behaviors cannot be expected from all MTSs or in all circumstances.

Our findings and theoretical implications also have applications for MTS training. Team training literature suggests that teams must be trained, not only on their task work, but also on their teamwork (e.g., Salas et al., 2001); MTS training should additionally include focus upon system-level work (Shuffler and Carter, 2018). This system-level focus should involve attention to different behavioral cycles that component teams might entrain, particularly in response to triggers for adaptation. That is, these findings highlight the importance of nuanced training tailored to both MTS attributes (i.e., boundary status and goal type) and situation type (e.g., contingency planning). For example, if external MTSs are more likely to return to alignment-focused cycles and over-rely on within-team behaviors, contingency training should include protocols or checklists for ensuring ongoing between-team interaction to ensure focus is maintained on the system’s superordinate goal, particularly in times of stress or crisis.

Finally, we suggest that the nature and structure of MTS training include particular attention to the nature of the MTS as a system of interdependent component teams who achieve their shared goal through careful balancing of efforts toward their proximal goals. That is, MTS training should not only focus on component teams’ efforts toward achieving their proximal goals *via* within-team behaviors, but also facilitate opportunity to train within-team alignment behaviors and, perhaps most importantly, between-team behaviors that are necessary for successful MTS performance.

### Limitations

Like all empirical studies, ours is limited by the nature and quality of the data. We were specifically limited by the availability and accessibility of the information pertaining to each case of MTS failure. Though we systematically applied rigorous and consistent criteria to all cases that were included to ensure that our findings were as generalizable as possible, this simultaneously served to limit the cases that were retained. Notably, sufficient detail on intellectual MTSs was particularly difficult to attain; as such, the data set only contains five cases of intellectual MTSs. This limitation is highlighted by the fact that all five intellectual cases are also all external MTSs, meaning no internal intellectual MTSs were represented in our findings. This may also be true of other combinations of attributes that were of lesser interest in this particular study and presents a path forward for future studies to target examinations on additional MTS attribute profiles.

Similarly, though we have many contexts represented among the cases, they are unequally proportioned. The most represented context is commercial flight with 12 cases, and the least represented are science MTSs with only one case. As such, readers should use caution when applying the results and using the themes presented as givens. Further research is needed to determine whether the findings are skewed by the underrepresentation of particular MTSs contexts that, if more readily available for inclusion in studies, such as ours, might allow for further refinement of the boundary conditions of the performance mechanisms presented herein. Specifically, further study is needed on intellectual MTSs and especially internal ones.

Even after passing our inclusion criteria, the nature of the cases, the sources used, and the information gleaned from them varied. For example, the black box transcripts available in NTSB investigative reports lent second-by-second detailed timelines of flight cases from a single source. For other cases, such as the Zano business failure, details of events were aggregated from several investigative journalist-written articles published on publicly available platforms (e.g., Medium.com; Harris, 2016). The variance in source type allowed for a more varied set of cases and, therefore, more generalizable findings; however, this inherently limited the variance among the source materials. Until certain types of MTSs (i.e., business context) and their performance are thoroughly, officially, and/or systematically documented, MTS research will continue to face insurmountable variance in cross-context comparison.

Relatedly, it should be noted that we looked at cases of failed MTS performance within the bounds of the action phase of a single performance episode. However, as the source documents frequently indicated, causes of failure are rarely singular or immediate. Because we examined behaviors within the action phase where the failure occurred, our findings may only describe behavior patterns of the teams during this period. We cannot speak to causes of failure that may have originated in the transition phase of performance or other causes that may have impacted failure prior to the action phase of the particular performance episode that was examined. Similarly, because we only looked at cases of failed performance, the findings presented may not hold for MTSs who experience near misses or successful responses to triggers, as we do not have comparative examples of successful MTS adaptive performance. Though we did compare the patterns in our cases to those described in certain, available SOP manuals, these presented the limitation of, naturally, only including standard operations. Many of the cases and contexts, particularly those of relatively unique or novel situations (e.g., the Willow Creek Community Church scandal response case) do not have standard operations whatsoever. This limitation presents an opening for future research, particularly field work, to pursue a deeper understanding of these types of MTSs and their performance.

### Future Directions

In addition to the openings for future research described above, our findings set up an opportunity for continued work to delve deeper into the mechanisms and impacts of entrainment as a phenomenon inter-related with MTS processes and performance. For example, additional research might consider the differing behavioral patterns of MTSs that fail to adjust compared to those that fail to adapt (see Torres et al., 2021, for a discussion of surface adjustment versus strategic/operational adaptation). If, as is described above, the MTS’s attributes impact the behavior patterns of its component teams, so too might the situational attributes, particularly as they change over time and in ways that threaten goal accomplishment. Finally, as a contrast to this study exploring the nature of failed cases, future researchers should consider patterns of successful and resilient MTSs. Insights into effective MTS operations, not just in typical circumstances, but in efforts to overcome triggers for adaptation, may inform how entrainment might be further leveraged as a framework to optimize the system as a whole.

## Conclusion

This study delves deeper into the nature of multilevel coordination interactions for MTSs across various contexts. Our findings highlight the importance of this knowledge for mitigating and preventing the large-scale harm that results from the failed performance of these systems. Our aim is that these findings are applied by organizations, practitioners, and researchers alike to advance the field and prepare the members of these systems to respond to the changes and challenges they face more readily and ably.

## Data Availability Statement

The raw data supporting the conclusions of this article will be made available by the authors, without undue reservation.

## Author Contributions

LC, ET, and SZa: study conception and design and draft manuscript preparation. LC, ET, SZh, and KH: data collection. LC, ET, SZa, SZ, KH, DW, CR, and JZ: analysis and interpretation of results. All authors contributed to the article and approved the submitted version.

## Conflict of Interest

The authors declare that the research was conducted in the absence of any commercial or financial relationships that could be construed as a potential conflict of interest.

## Publisher’s Note

All claims expressed in this article are solely those of the authors and do not necessarily represent those of their affiliated organizations, or those of the publisher, the editors and the reviewers. Any product that may be evaluated in this article, or claim that may be made by its manufacturer, is not guaranteed or endorsed by the publisher.
